# Intestinal Complication With Myxomatous Mitral Valve Diseases in Chihuahuas

**DOI:** 10.3389/fvets.2021.777579

**Published:** 2021-11-23

**Authors:** R. Araki, K. Iwanaga, Kazunori Ueda, M. Isaka

**Affiliations:** ^1^Yokohama Yamate Dog & Cat Medical Center, Yokohama, Japan; ^2^Tokyo Veterinary Cardiology Center, Fukazawa, Japan; ^3^Department of Small Animal Clinical Sciences, School of Veterinary Medicine, Rakuno Gakuen University, Ebetsu, Japan

**Keywords:** Chihuahuas, D-lactate, intestinal complication, intestinal fatty acid-binding protein, intestinal mucosal injury markers, myxomatous mitral valve disease

## Abstract

The effects of cardiac disease on the intestine have been reported in humans but not in dogs. We investigated the effects of myxomatous mitral valve disease (MMVD), which is capable of causing congestion and tissue hypoperfusion, on the intestine in Chihuahuas, a breed frequently encountered in clinical practice as the preferred breed for MMVD. In this study, 69 Chihuahuas were divided into four groups based on echocardiography and chest radiography: 19 healthy Chihuahuas (H) and 50 Chihuahuas with MMVD classified according to the ACVIM consensus (stage B1, B2, C/D). In all the cases, serum intestinal fatty acid-binding protein (I-FABP) and D/L-lactate concentrations, markers of intestinal mucosal injury, were measured. I-FABP was significantly higher in stage C/D Chihuahuas than in other groups (*p* < 0.05), and stage B2 was significantly higher than H (*p* < 0.05). D-lactate was significantly increased in stages B2 and C/D compared to H and stage B1 (*p* < 0.05). L-lactate was significantly higher in stage C/D Chihuahuas than in any other group (*p* < 0.05), and stage B2 was significantly higher than that in H and stage B1 (*p* < 0.05). Intestinal mucosal injury risk was significantly higher in Chihuahuas with heart failure due to MMVD, suggesting that the risk could increase with worsening heart disease. This is the first study to investigate the intestinal complications of MMVD, and further investigations a needed in the future.

## Introduction

The heart is a vital organ, and many associations between cardiovascular diseases and other organs, such as the liver (1) and kidney (2, 3), have been reported. Among these, the heart and intestine are recognized as being closely relatssed (1, 4, 5). In patients with heart failure, ischemia owing to hypoperfusion attributed to decreased cardiac output to the mesenteric arteries and intestinal mucosa (6), and intestinal edema owing to venous congestion (7) cause increased intestinal mucosal permeability. As a result, the lipotoxin (LPS) contained in the cell walls of gram-negative bacteria in the gut translocates into the blood (8), causing systemic inflammation and multi-organ damage (9–12). Myxomatous mitral valve disease (MMVD) is the most common cause of congestive heart failure in small dogs and is characterized by progressive atrioventricular valve degeneration (13). Prolonged heart failure can result in reduced blood flow in the body, causing ischemic damage to vital organs, such as the kidneys (14) and pancreas (15, 16). While the association between cardiac disease and the intestine has been widely reported in human medicine, it has not yet been studied and is still unknown in veterinary medicine.

Intestinal fatty acid-binding protein (I-FABP), which is a 15 kD protein responsible for the transport of long-chain fatty acids from the intestinal lumen (17, 18), is localized to the small intestinal mucosal epithelial cells. Since it is a relatively low-molecular weight, renally excreted, soluble cytoplasmic protein, it is rapidly released and translocated into the bloodstream when the small intestinal mucosal epithelial cells are injured (19, 20). I-FABP elevations have been observed in patients with intestinal diseases and systemic inflammatory response syndromes (21–25) and have been recognized as useful markers of intestinal mucosal injury (23, 24).

Lactate, which is an end product of anaerobic metabolism, is considered a biomarker for the diagnosis and prognosis of shock in humans and in veterinary medicine, and for detecting hypoperfusion (26). Lactate exists in the L- and D-body optical isomers. L- lactate is also produced by anaerobic metabolism associated with intestinal ischemia; however, it is not specific to the intestinal tract, since it increases in hypoxia related to all organs. In contrast, D-lactate is not produced in mammalian tissues but is produced by the methylglyoxal pathway and the enteric bacterial pathway; therefore, elevated D-lactate levels in the blood are considered to be of bacterial origin (26, 27); in humans, blood D-lactate is known to be elevated in patients with diabetes, infection, ulcerative colitis, intestinal surgery, and intestinal necrosis (28–30). D-lactate could be considered a specific marker for intestinal mucosal injury since it flows into the portal bloodstream when intestinal mucosal epithelial injury occurs owing to intestinal ischemia, and abnormal intestinal bacterial growth occurs (31). Hence, it is expected to be a potential intestinal mucosal injury marker (32–34).

Previous reports have used the lactulose-mannitol test and catheter- based right atrial pressure measurement to investigate intestinal permeability and venous congestion (7); however, reproducibility in dogs is difficult. In this study, we investigated the risk of intestinal mucosal injury using I-FABP and D/L-Lactate in healthy dogs and dogs with MMVD, limiting the study breed to clinically commonly encountered Chihuahuas. We hypothesized that intestinal mucosal injury could be a complication of MMVD in dogs and that the markers of intestinal mucosal injury would be significantly increased in dogs with MMVD.

## Materials and Methods

### Animals

This study was approved by the Rakuno Gakuen University, School of Veterinary Medicine Institutional Animal Care and Use Committee (approval No. VH19A10). All the cases were sampled between April 2019 and May 2020. Samples were collected at two institutions: Yokohama Yamate Dogs and Cats Medical Center and Tokyo Veterinary Cardiology Center. The sample was limited to Chihuahuas only, and included cases with MMVD or healthy dogs. Most of the healthy Chihuahuas visited the hospital for checkups and preventive medical care such as rabies vaccines. All Chihuahuas that met the inclusion criteria during the study period were included in the sample collection. The inclusion criteria were an adult dog, consent to the study, and absence of any urgent or fatal comorbidities. Patients with diseases secondary to MMVD (e.g., pulmonary hypertension or chronic kidney disease) were included; however, patients with other cardiac diseases (e.g., cardiac tumor or epicardial disease) were excluded. After obtaining the owner's consent, physical examination (weight and body condition score [BCS]), auscultation (presence and intensity of heart murmur), medical history, current medical history, medications, antibiotics, and the usual diet were recorded in all cases. In all the cases, chest radiography and echocardiography were performed to evaluate the heart. Based on the information obtained, healthy cases (H group) and cases with MMVD were classified into three groups according to the severity grade (stage B1, B2, and C/D) based on the ACVIM guidelines (35). Patients without cardiac disease were classified as healthy. We also performed systemic screening tests, including blood tests and radio/echography, and referred to past medical records to assess comorbidities. Serum samples were also collected to measure IFABP and L/D lactate concentrations by enzyme-linked immunosorbent assay (ELISA).

### Echocardiography and Chest Radiology

Echocardiography and chest radiography were performed in all dogs. Echocardiography was performed according to standard techniques (36), using Xario (TOSHIBA, probe: PST-50AT, 5 MHz) at the Yokohama Yamate Dogs and Cats Medical Center and Vivid e95 (GE Healthcare Japan, probe: 6S) at the Tokyo Veterinary Cardiology Center. Data were collected by two examiners; all cases were examined in a quiet room. Echocardiography included the subjective evaluation of the valve structure and function; mitral regurgitation (MR) jets on color Doppler examination in four-lumen cross-sectional images, left atrium to aorta ratio (LA/Ao) at diastole, left ventricular septal wall thickness at diastole (IVSd), left ventricular posterior wall thickness at diastole (LVPWd), left ventricular internal diameter at diastole (LVIDd), normalized left ventricular internal diameter (LVIDDN) (37), left ventricular internal diameters in systole (LVIDs), fractional shortening (FS), left ventricular ejection fraction (LVEF), and left ventricular inflow velocity waves (Evel, Avel) were evaluated and recorded.

Chest radiographs were taken at maximal right lateral inspiration for the presence of pulmonary edema and for recording the vertebral heart scale (VHS) and vertebral left atrial score (VLAS). The VHS and VLAS were measured using a previously described method (38, 39).

The disease severity was determined as cardiac enlargement (Stage B2) at LA/Ao>1.6 and LNIDDN>1.7 using ACVIM guidelines (35). Although the normal references of VHS and VLAS reportedly vary depending on the breed of dog, a previous report (40) reveals the standard for Chihuahuas; hence, we followed that report and used VHS > 10.0 v and VLAS > 1.8 v as the standard for cardiac enlargement. Patients with current or past pulmonary edema were defined as stage C; patients with refractory pulmonary edema were defined as stage D.

### Serum Samples

Blood samples were collected in all cases. Blood was collected from the jugular vein or the external saphenous vein. The blood sample was placed in a serum separator tube, meticulously inverted, and mixed. The clots were left at room temperature for 15–30 min until the coagulation was complete and then centrifuged at 1,100 g for 10–15 min following clot formation. The serum obtained by centrifugation was transferred to microtubes and stored at −80°C for I-FABP, L-lactate, and D-lactate measurement using ELISA.

### Enzyme-Linked Immuno Sorbent Assay

I-FABP levels were measured using the canine intestinal fatty acid binding protein ELISA kit (Bioassay Technology Laboratory), as in the previous study (41). The assay was performed according to the manufacturer's recommendations. The serum was allowed to warm up naturally at room temperature, and the serum was then diluted. Serum samples and ELISA reagents were placed in each well and incubated at 37°C for 1 h. The plate was washed five times and then incubated at 37°C for 10 min with substrate solution added, followed by the addition of stop solution and color development, and absorbance measurement at 450 nm. For the controls, we measured what was included in the manufacturer's manual. Measurements were taken in duplicate, and the cut-off was 2 standard deviations. No samples were below the lower detection limit or above the upper detection limit.

D/L-lactate was measured using the EnzyChoromTM D-lactate assay kit (ECLC-100) (BioAssay Systems) and the EnzyChoromTM L-Lactate Assay Kit (EDLC-100) (BioAssay Systems). ECLC-100 and EDLC-100 were not used for antibody measurement. These kits were used for all animals. The assay was performed according to the manufacturer's recommendations. Serum was diluted 2- fold with dH2O before the assay, and the absorbance was measured at 565 nm.

### Statistical Analysis

Data were computed into a spreadsheet, and statistical analysis was performed using SPSS software (SPSS statistics ver 24.0 IBM Japan, Ltd., Tokyo, Japan). Histograms were made for continuous variables to assess normality and equivariance. There were no significant deviations as a parametric method for all variables, and the results were judged to be within a tolerable range. ELISA (I-FABP, D-lactate, L- Lactate) results were subjected to a one-way ANOVA using the Tukey– Kramer *post-hoc* test. Results are presented as mean ± standard deviation. Differences were considered significant at *P* < 0.05.

## Results

### Animals

We collected a sample of 69 cases, ranging in age from 3 to 12 years, which were divided into four groups: H (*n* = 19), B1 (*n* = 21), B2 (*n* = 15), and C/D (*n* = 14) (Table 1). Of the 14 cases in Group C/D, three samples were collected on presentation to the hospital in a hypoxemic crisis of fulminant pulmonary edema. The remaining 11 cases were sampled while stable on medication at a scheduled recheck. No cases were excluded after sample collection. Group H had a predominantly lower mean age compared to the other groups; however, no age difference was observed in the other groups. One case each in groups B1 and C/D was on antimicrobial medication. They were administered erythromycin and enrofloxacin, respectively. The following comorbidities were observed in each group. Group H: canine atopic dermatitis (CAD) (*n* = 2), chronic kidney disease (CKD) (*n* = 1), epileptic seizures (*n* = 1), and food allergy (*n* = 1). Group B1: CAD (*n* = 1), cholelithiasis (*n* = 1), hypoadrenocorticism (*n* = 1), and hypothyroidism (*n* = 2). Group B2: CAD (*n* = 1), CKD (*n* = 2), diabetes mellitus (*n* = 1), tracheal collapse (*n* = 2), and hypothyroidism (*n* = 1). Group C/D: CKD (*n* = 7) and tracheal collapse (*n* = 2). Owing to worsening cardiac disease, many patients were taking cardiac medications, and all the patients in Group C/D were taking angiotensin-converting enzyme inhibitors (ACI) and pimobendan. The three patients in Group B2 on loop diuretics (furosemide: 1.1 ± 0.6 mg/kg/day) had no history of heart failure and, were being prescribed for cough reduction. Nine patients in Group C/D were receiving loop diuretics (torasemide: 0.33 ± 0.23 mg/kg/day).

**Table 1:** Patient characteristics of healthy Chihuahuas (H) and Chihuahuas with myxomatous mitral valve disease divided into three groups (B1, B2, and C/D).

	**H (*n =* 19)**	**B1 (*n =* 21)**	**B2 (*n =* 15)**	**C/D (*n =* 14)**
Age (years)	7.3 ± 2.6	11.1 ± 2.5	12.0 ± 2.4	11.8 ± 1.9
Sex (M/F)	9/10	11/10	6/9	8/6
Male/Casted male	0/9	2/9	2/4	3/5
Female/Spayed female	0/10	0/10	1/8	1/5
BW (kg)	3.1 ± 1.2	2.8 ± 1.0	3.0 ± 0.9	3.1 ± 1.0
Antibiotics	Nothing (*n =* 19)	Nothing (*n =* 20) Erythromycin (*n =* 1)	Nothing (*n =* 15)	Nothing (*n =* 13) Enrofloxacin (*n =* 1)
Comorbidities	CAD (*n =* 1)	CAD (*n =* 1)	CAD (*n =* 1)	CKD (*n =* 7)
	CKD (*n =* 1)	Cholelithiasis (*n =* 1)	CKD (*n =* 2)	Tracheal collapse (*n =* 2)
	Epileptic seizures (*n =* 1)	Hypoadrenocorticism (*n =* 1)	Diabetes mellitus (*n =* 1)	
	Food allergy (*n =* 1)	Hypothyroidism (*n =* 2)	Hypothyroidism (*n =* 1)	
			Tracheal collapse (*n =* 2)	
**Medicine**
ACE-I	*n =* 1	*n =* 2	*n =* 10	*n =* 14
Pimobendan	Nothing	*n =* 1	*n =* 13	*n =* 14
Loop-diuretics	Nothing	Nothing	*n =* 3 (furosemide: 1.1 ± 0.6 mg/kg/day)	*n =* 9(trasemide: 0.33 ± 0.23 mg/kg/day)

*Data are presented as mean ± standard deviation (SD)*.

*BW, body weight; BCS, body condition score; CAD, canine atopic dermatitis; CKD, chronic kidney disease; ACE-I angiotensin-converting enzyme inhibitor*.

### Echocardiography and Chest Radiography

Echocardiography, VHS, and VLAS findings are presented in Table 2. LVIDd was higher in groups B2 and C/D than in groups H and B1; however, there was no significant difference between groups B2 and C/D. The E wave also increased with severity, with group C/D having the highest increase. VHS and VLAS were also higher with worsening cardiac disease, with Group C/D having higher values than the other groups.

**Table 2:** Echocardiographic and radiographic data in healthy Chihuahuas (H) and Chihuahuas with myxomatous mitral valve disease divided into three groups (B1, B2, and C/D).

	**H (*n =* 19)**	**B1 (*n =* 21)**	**B2 (*n =* 15)**	**C/D (*n =* 14)**	***P*-value for all**
LA (mm)	13.2 ± 2.2	15.2 ± 2.7	23.3 ± 6.1^b^, ^d^	32.0 ± 7.4^c^, ^e^, ^f^	<0.001
Ao (mm)	9.41 ± 1.0	10.2 ± 1.2	10.9 ± 1.3^b^	10.5 ± 1.3^c^	0.005
LA/Ao	1.4 ± 0.2	1.5 ± 0.2	2.1 ± 0.5^b^, ^d^	3.1 ± 0.9^c^, ^e^, ^f^	<0.001
IVSDd(mm)	5.2 ± 1.2	5.2 ± 0.8	4.6 ± 0.9	4.4 ± 1.2	0.062
LVPWd(mm)	5.4 ± 0.9	5.6 ± 1.0	5.1 ± 0.8	5.1 ± 1.2	0.292
LVIDd(mm)	17.0 ± 2.6	19.1 ± 3.4	25.5 ± 3.4^b^, ^d^	27.5 ± 4.1^c^, ^e^	<0.001
LVIDs(mm)	10.8 ± 2.6	12.0 ± 2.2	14.1 ± 2.3^b^	15.5 ± 3.6^c^, ^e^	<0.001
LVIDDN	1.7 ± 0.2	1.8 ± 0.1	1.9 ± 0.1^b^	1.9 ± 0.2^c^	<0.001
E vel(m/s)	0.6 ± 0.1	0.7 ± 0.2	0.9 ± 0.2^b^	1.3 ± 0.5^c^, ^e^, ^f^	<0.001
A vel(m/s)	0.6 ± 0.2	0.8 ± 0.2^a^	1.0 ± 0.2^b^	0.8 ± 0.3	<0.001
MR vel(m/s)	nothing	5.7 ± 0.6	5.4 ± 0.5	5.3 ± 0.6	0.286
FS(%)	35.7 ± 6.3	36.0 ± 6.7	38.8 ± 12.9	44.4 ± 11.0^c^	0.037
LVEF(%)	68.3 ± 8.4	67.5 ± 7.6	72.0 ± 9.0	72.6 ± 11.7	0.338
VHS(v)	9.2 ± 0.9	9.4 ± 0.8^a^	10.6 ± 0.7^b^, ^d^	11.5 ± 1.2^c^, ^e^, ^f^	<0.001
VLAS(v)	2.0 ± 0.2	2.3 ± 0.3^a^	3.1 ± 0.3^b^, ^d^	3.8 ± 0.4^c^, ^e^, ^f^	<0.001

*Data are presented as mean ± standard deviation (SD)*.

*LA, left atrium; Ao, aorta; LA/Ao, maximal diastolic left atrial body-to-aortic root diameter ratio in the right, parasternal, short-axis view of the heart base; IVSTd, interventricular septum thickness in diastole; LVPWd, left ventricular posterior wall thickness in diastole; LVIDd, left ventricular internal diameter in diastole; LVIDs, left ventricular internal diameter in systole; LVIDDN, left ventricular internal diameter in diastole, indexed to the bodyweight; E vel, early wave velocity; A vel, atrium wave velocity; MR vel, mitral regurgitation velocity; FS, shortening fraction; LVEF, left ventricular ejection fraction; VHS, vertebral heart score; VLAS, vertebral left atrial size*.

*P-value for all: Results of the overall ANOVA F-test*.

*Pairwise comparisons were made using the Tukey-Kramer test:*

a *Statistically significant difference between H and B1 (P <0.05)*.

b *Statistically significant difference between H and B2 (P <0.05)*.

c *Statistically significant difference between H and C/D (P <0.05)*.

d *Statistically significant difference between B1 and B2 (P <0.05)*.

e *Statistically significant difference between B1 and C/D (P <0.05)*.

f *Statistically significant difference between B2 and C/D (P <0.05)*.

### Serum I-FABP, D-and L-Lactate Concentrations

**Figure 1:**
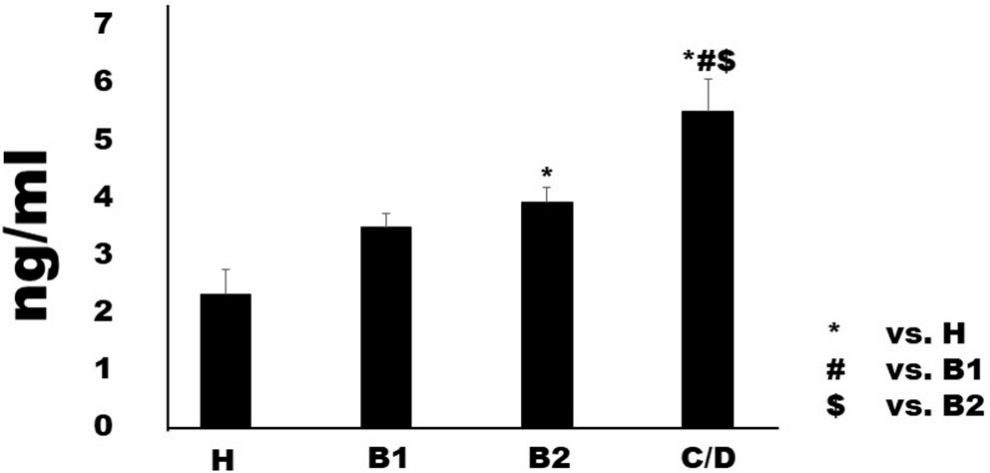
Serum concentrations of intestinal fatty-acid binding protein in healthy Chihuahuas (H) and Chihuahuas with myxomatous mitral valve disease divided into three groups (B1, B2, and C/D). One-way ANOVA with *post hoc* Turkey-Kramer test. Significant difference was considered *P* < 0.05.

**Figure 2:**
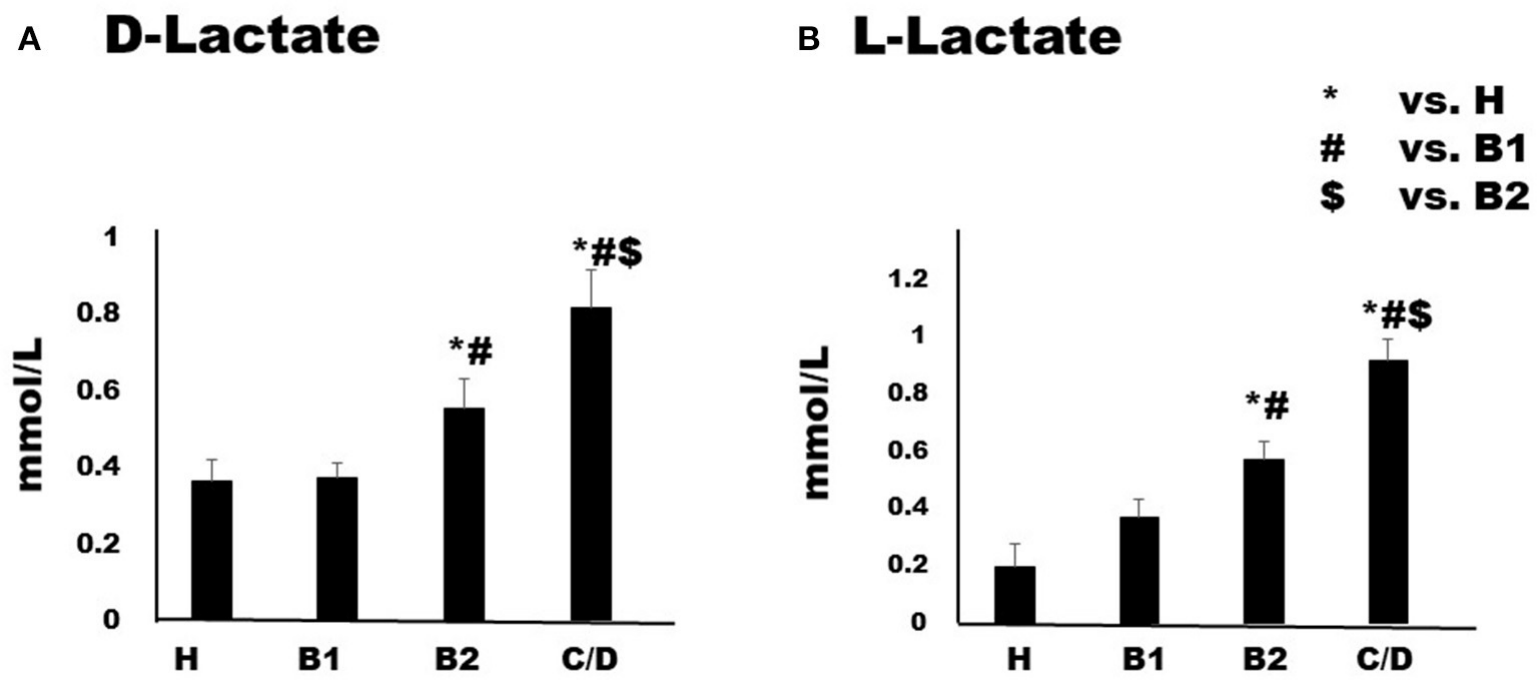
Serum concentrations of D-Lactate **(A)** and L-Lactate **(B)** in healthy Chihuahuas (H) and Chihuahuas with myxomatous mitral valve disease divided into three groups (B1, B2, and C/D). One-way ANOVA with *post hoc* Turkey-Kramer test. Significant difference was considered *P* < 0.05.

Serum I-FABP concentration in Chihuahuas with MMVD increased in proportion to MMVD severity (Figure 1). Although Group B1 (2.32 ± 0.43 ng/ml) was not significant difference compared to Group H (2.32 ± 0.43 ng/ml), Group B2 (3.93 ± 0.25 ng/ml) showed predominantly higher serum levels than group H. Group C/D (5.50 ± 0.57 ng/ml) showed predominantly higher serum concentrations than the other groups. Serum D-lactate and L-lactate concentrations are shown in Figures (Figures 2A,B). Considering D-lactate, group B2 (0.55 ± 0.08 mmol/L) had increased serum concentrations compared to groups H (0.36 ± 0.06 mmol/L) and B1 (0.37 ± 0.04 mmol/L). Group C/D (0.82 ± 0.09 mmol/L) showed an overall increase in serum concentration compared to the other groups. The concentrations of serum L-lactate (H: 0.2 ± 0.08 mmol/L; B1: 0.37 ± 0.07 mmol/L; B2: 0.57 ± 0.06 mmol/L; 0.92 ± 0.07 mmol/L) were shown as the same phenomenon as the results of D-lactate.

## Discussion

The main objective of our study was to clarify the relationship between MMVD and intestinal mucosal injury using intestinal mucosal injury markers such as I-FABP and D-lactate.

We found that serum I-FABP, D-lactate, and L-lactate levels were increased in Chihuahuas with heart failure attributed to MMVD, and there was a certain correlation between worsening heart disease and increased I-FABP, D-lactate, and L-lactate levels. I-FABP was significantly higher in group B2 than in group H, and in group C/D more than in any other group. D-lactate was significantly increased in the groups with cardiac enlargement (B2, C/D) than in groups without cardiac enlargement (H, B1). L-lactate was significantly higher in group B2 than in groups H and B1, and the C/D group showed an increase over the other groups. These results suggest that the risk of intestinal mucosal injury could increase with worsening heart disease, and dogs with heart failure (Group C/D) had a significantly higher risk of intestinal mucosal injury than dogs without heart failure (Group H, B1, B2).

Kitai et al. (42) reported that the cut-off value of I-FABP in human patients with heart failure was 853 pg/ml, suggesting that a case group with >853 pg/ml could have a poor prognosis with a short survival time. In the present study, I-FABP was significantly increased in Group C/D to 5.5 ( ± 0.6) ng/ml, and the difference in value was the difference between humans and dogs. In the future, if I-FABP is studied in dogs and a cut-off value can be established, it could become a new prognostic factor for heart failure in dogs. L-Lactate was significantly increased in heart failure, which was consistent with previous reports (43). There are no reports on the association between D- lactate levels and heart failure. However, the intestinal-specific increase in D-lactate suggests intestinal mucosal injury, as reported by Montagna et al. (33). The significant increase in D-lactate levels in dogs with heart failure in this study suggests that dogs with heart failure are at risk for intestinal mucosal injury. A study reported by Venn et al. (31) demonstrated that D-lactate was significantly elevated in dogs with parvoviral enteritis; therefore, D-lactate possesses the potential for use as a biomarker in dogs. However, Memet et al. (32) reported that D/L-Lactate is often increased at the end of severe intestinal injury and is considered inferior to I-FABP as a marker of intestinal mucosal injury. In this study, D/L-Lactate was similar to I-FABP; however, further studies are warranted to determine its usefulness in dogs.

LA/Ao > 1.7 and E vel > 1.2 m/s are reportedly poor prognostic factors in MMVD (44); in the present study, the values of LA/Ao and E vel were significantly higher in the C/D group than in the other groups, consistent with the reports of Borgarelli et al. I-FABP and D-lactate levels increased in proportion to MMVD severity, suggesting a possible correlation between intestinal mucosal injury and important echocardiographic parameters that indicate the prognosis of MMVD, such as LA/Ao and E vel. The more severe the MMVD, the greater the increase and decrease in FS and LVEF, respectively. Although no significant difference was found in this study, both FS and LVEF increased with worsening MMVD, which may be due to the influence of the many cases in Group B2 and all the cases in C/D who were taking pimobendan. Kanno et al. suggested that pimobendan administration increased FS and LVEF (45). It is difficult to relate cardiac contractile function to intestinal mucosal injury in this study. Kitai et al. (42) reported no association between the increased I-FABP levels and echocardiographic parameters. This is because, in this study, the collection of patients (healthy dogs to dogs with severe heart failure) was blinded, and subsequently classified and measured for severity. However, Kitai et al. (42) limited their study to patients with heart failure. In addition, the heart failure in humans studied by Kitai et al. (42) is due to cardiomyopathy, which differs in pathology from the heart failure due to MMVD in dogs in this study. Therefore, these results were inconsistent with our study owing to differences in the original population and pathology.

Performing VHS and VLAS is easier compared to echocardiography; VLAS, in particular, significantly correlates with LA/Ao measured by echocardiography (39). The more severe the MMVD, the greater the increase in VHS and VLAS; dogs with heart failure showed a significant increase compared to the other groups, suggesting a correlation between I-FABP and D/L-Lactate, which increased with MMVD severity. A correlation between VHS, VLAS, and intestinal mucosal injury markers, proven by further research, is desirable since it would result in a simpler test for determining the risk intestinal mucosal injury. However, the accuracy of VHS and VLAS is inferior to that of echocardiography since it depends on many factors, such as canine body position, radiographic technique, and the presence or absence of concomitant chest abnormalities. Therefore, the practicality of VHS and VLAS as a test to determine the risk of intestinal mucosal injury is uncertain.

Our study had certain limitations. First, there was a lack of uniformity in the diet of the patients. The effects of diet on the intestine and health in dogs have been reported (46, 47), and differences in diet could have affected the results of our study. Second, previous reports have used pulmonary artery catheterization to measure right atrial pressure to investigate venous stasis (42) and the lactulose-mannitol test to test intestinal permeability (7). Although it is a highly credible test, it is not an easy test to perform in veterinary medicine and does not increase the credibility of the results of this study. Third, the presence or absence of concomitant diseases may have affected the results. Group C/D had more cases using diuretics to treat cardiac disease and also showed a mild to severe increase in the renal values. The impact of an increased renal panel on the results of this study should be considered. Other gut bacteria are deeply involved in the relationship between the heart and the gut (48); however, in this study, we did not assess gut bacteria, blood LPS levels, or intestinal mucosa. Further studies are warranted to clarify the relationship between heart disease and intestines in dogs.

## Conclusions

In human medicine, many reports exist on the effects of cardiac disease on the gut; however, no studies have been reported in dogs. This study showed that intestinal mucosal injury markers such as I-FABP and D-Lactate increased with the severity of MMVD in Chihuahuas and that Chihuahuas with MMVD who had heart failure were at particular risk for intestinal mucosal injury. This study is the first to investigate the relationship between heart disease and intestinal mucosa in Chihuahuas and demonstrate a potential relationship between MMVD morbidity and intestinal mucosal cell injury.

## Data Availability Statement

The raw data supporting the conclusions of this article will be made available by the authors, without undue reservation.

## Ethics Statement

The animal study was reviewed and approved by the Rakuno Gakuen University, School of Veterinary Medicine Institutional Animal Care and Use Committee (Approval No. VH19A10). Written informed consent was obtained from the owners for the participation of their animals in this study.

## Author Contributions

Conceptualization, methodology, formal analysis, writing review, editing, visualization, and supervision: MI. Software, validation, data curation, and writing-original draft preparation: RA. Investigation: RA and KI. Project administration: KU and MI. All authors have read and agreed to the published version of the manuscript.

## Conflict of Interest

The authors declare that the research was conducted in the absence of any commercial or financial relationships that could be construed as a potential conflict of interest.

## Publisher's Note

All claims expressed in this article are solely those of the authors and do not necessarily represent those of their affiliated organizations, or those of the publisher, the editors and the reviewers. Any product that may be evaluated in this article, or claim that may be made by its manufacturer, is not guaranteed or endorsed by the publisher.
